# ACT-Based Online Intervention for Obsessive-Compulsive Traits, Anxiety, and Depression in Medical Students: A Pilot Study

**DOI:** 10.1192/bjo.2025.10135

**Published:** 2025-06-20

**Authors:** Athanasios Hassoulas, Rachel McCrum, Angelina Patel, Khaleda Rashid, Prasanna Vaidhaya

**Affiliations:** Cardiff University School of Medicine, Cardiff, United Kingdom

## Abstract

**Aims:** It is estimated that the prevalence of obsessive-compulsive disorder (OCD) in the UK is 2–3%, with a lifetime prevalence of anxiety and/or depression being reportedly much higher (estimated at 20–25%). Prevalence rates within medical students have, however, been reported by numerous studies and systematic reviews to exceed those identified in the general population. The primary aim of the current pilot study was to investigate the efficacy of an Acceptance and Commitment Therapy (ACT)-based online intervention in reducing symptom severity within a sample of medical students screened for OCD, anxiety and depressive traits and symptoms. The hypothesis was that the ACT-based online intervention would reduce symptom severity in participants.

**Methods:** Participants were recruited from Cardiff University’s School of Medicine and were required to complete baseline screening measures for OCD, anxiety, and depressive traits using the Obsessive-Compulsive Inventory-Revised version (OCI-R), the State Trait Anxiety Inventory (STAI), and the Beck Depression Inventory-Revised version (BDI-II), respectively. A total of 11 medical students were recruited following screening and completed two parts of an online ACT-based intervention over a two-week period. Participants were required to complete the same measures post-intervention and were debriefed following their participation. Ethical approval for this study was provided by the university’s School of Medicine Research Ethics Committee.

**Results:** Data collated was analysed to measure for any differences pre and post intervention. A reduction in OCD symptom severity was reported, however with findings approaching significance (z=−1.362, p>0.05). A significant difference in pre and post intervention scores was not revealed for depressive traits, however a significant reduction in anxiety was revealed using the STAI measure pre and post intervention, (z=−2.59, p<0.01).

**Conclusion:** The findings of the current pilot highlight the efficacy of our ACT-based online intervention in significantly reducing anxiety in a cohort of medical students. Whilst a small sample was recruited for this pilot, the results warrant further investigation and consideration by university student services in adopting the intervention for students.

